# Mouse embryonic stem cells with a multi-integrase mouse artificial chromosome for transchromosomic mouse generation

**DOI:** 10.1007/s11248-015-9884-6

**Published:** 2015-06-09

**Authors:** Yuki Yoshimura, Kazuomi Nakamura, Takeshi Endo, Naoyo Kajitani, Kanako Kazuki, Yasuhiro Kazuki, Hiroyuki Kugoh, Mitsuo Oshimura, Tetsuya Ohbayashi

**Affiliations:** Division of Laboratory Animal Science, Research Center for Bioscience and Technology, Tottori University, 86 Nishi-cho, Yonago, Tottori 683-8503 Japan; Department of Biomedical Science, Graduate School of Medical Sciences, Institute of Regenerative Medicine and Biofunction, Tottori University, 86 Nishi-cho, Yonago, Tottori 683-8503 Japan; Organizations for Tottori Industrial Promotion, 7-5-1 Wakabadaiminami, Tottori, Tottori, 689-1112 Japan; Chromosome Engineering Research Center (CERC), Tottori University, 86 Nishi-cho, Yonago, Tottori 683-8503 Japan; Department of Experimental Animals, Interdisciplinary Center for Science Research, Organization for Research, Shimane University, 89-1 Enya-cho, Izumo, Shimane 693-8501 Japan

**Keywords:** Transchromosomic mice, Mouse artificial chromosome, Gene delivery

## Abstract

The mouse artificial chromosome (MAC) has several advantages as a gene delivery vector, including stable episomal maintenance of the exogenous genetic material and the ability to carry large and/or multiple gene inserts including their regulatory elements. Previously, a MAC containing multi-integration site (MI-MAC) was generated to facilitate transfer of multiple genes into desired cells. To generate transchromosomic (Tc) mice containing a MI-MAC with genes of interest, the desired genes were inserted into MI-MAC in CHO cells, and then the MI-MAC was transferred to mouse embryonic stem (mES) cells via microcell-mediated chromosome transfer (MMCT). However, the efficiency of MMCT from CHO to mES cells is very low (<10^−6^). In this study, we constructed mES cell lines containing a MI-MAC vector to directly insert a gene of interest into the MI-MAC in mES cells via a simple transfection method for Tc mouse generation. The recombination rate of the GFP gene at each attachment site (FRT, PhiC31attP, R4attP, TP901-1attP and Bxb1attP) on MI-MAC was greater than 50 % in MI-MAC mES cells. Chimeric mice with high coat colour chimerism were generated from the MI-MAC mES cell lines and germline transmission from the chimera was observed. As an example for the generation of Tc mice with a desired gene by the MI-MAC mES approach, a Tc mouse strain ubiquitously expressing Emerald luciferase was efficiently established. Thus, the findings suggest that this new Tc strategy employing mES cells and a MI-MAC vector is efficient and useful for animal transgenesis.

**Electronic supplementary material:**

The online version of this article (doi:10.1007/s11248-015-9884-6) contains supplementary material, which is available to authorized users.

## Introduction

Transgenic (Tg) animals are powerful tools for investigating gene function (Olive et al. 2004), and gene therapy (Shi et al. 2014), and can function as animal models of human diseases (Masliah et al. 2000). Tg animals have been successfully generated by microinjection of genes of interest into the pronuclei of fertilised eggs, allowing the generation of transgenic animals in many species including mouse, rat, cow and pig. However, the copy number and location of a transgene inserted into the host genome typically cannot be controlled because it is a random event. Furthermore, expression of the gene of interest is susceptible to positional effects. To overcome this problem, the knock in (KI) of one copy of a desired gene at a defined locus such as the *Rosa26* or *Hprt* gene region by homologous recombination in mouse embryonic stem (mES) cells has been used to generate Tg mice stably expressing the gene (Soriano 1999; Yang et al. 2009). However, in the KI approach, Mb-sized and multiple different genes cannot be transferred to a single locus.

Human artificial chromosomes (HACs) and mouse artificial chromosomes (MACs) exhibit several important characteristics desirable of an ideal gene delivery vector, including stable episomal maintenance that avoids insertional mutations, and the capacity to carry large genomic loci with their regulatory elements. This facilitates physiological regulation of the introduced gene in a manner similar to that of the native chromosome (Ren et al. 2006; Oshimura et al. 2013). Transchromosomic (Tc) technology utilising HACs or MACs has been used for the generation of animals containing Mb-sized segments of the desired gene (Kuroiwa et al. 2009; Kazuki et al. 2013a; Miyamoto et al. 2014).

**Fig. 1:**
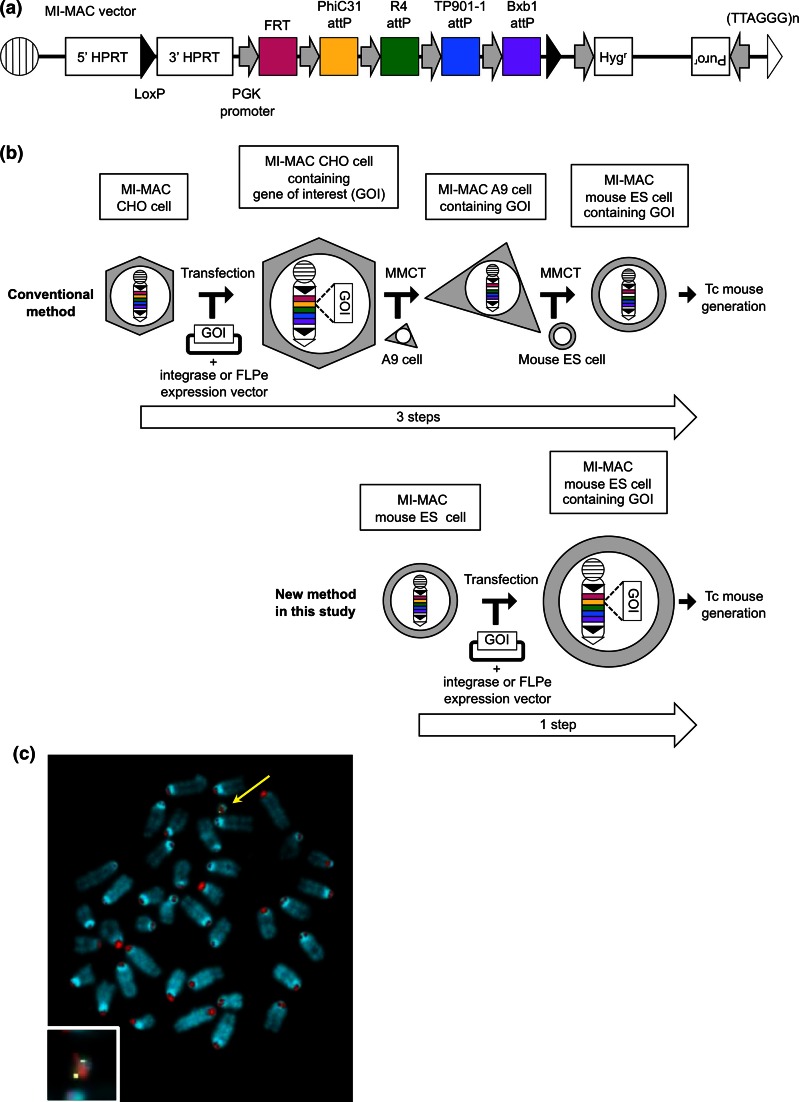
Establishment of multi-integrase mouse artificial chromosome mouse embryonic stem (MI-MAC mES) cells. **a** Schematic of MI-MAC. **b** Comparison of two methods to generate Tc mice. In the conventional method, two or three steps are needed, and one or two round(s) of MMCT are needed. In the new method in this study, only one step (simple transfection, without MMCT) is needed. **c** Fluorescence in situ hybridisation (FISH) analysis of MI-MAC mES cell line. Digoxigenin-labelled mouse minor satellite (*red*) and Biotin-labelled multi-integrase (MI) platform (*green*) were used to detect mouse chromosomes and MI platform on the MAC, respectively. The *inset* shows an enlarged image of the MI-MAC (*arrow*)

Integrases are powerful tools used to insert a gene of interest in vitro (Yamaguchi et al. 2011) and in vivo (Tasic et al. 2011) by site-specific recombination between appropriate *attB* and *attP* sites. Yamaguchi et al. reported the construction of a multi-integrase (MI) system on HACs to validate site-specific recombination by PhiC31 (Kuhstoss and Rao 1991), R4 (Matsuura et al. 1996), TP901-1 (Christiansen et al. 1996), and Bxb1 (Mediavilla et al. 2000) integrases or FLPe recombinase (Rodríguez et al. 2000) in Chinese hamster ovary (CHO) cells (Yamaguchi et al. 2011). These integrases conferred higher site-specific recombination efficiency (39.3–96.8 %) in CHO cells than FLPe recombinase (17.2 %). Homogeneous transgene expression was observed in this MI system but not in the random integration system. Takiguchi et al. reported that the MI-MAC vector was constructed in the same way as the MI-HAC vector because the retention rates of the HAC vector were not uniform throughout tissues of Tc mice, and in particular are very low in haematopoietic cells (Takiguchi et al. 2012) (Fig. 1a). If an MI-HAC/MAC vector is transferred into mES cells, Tc mice expressing multiple target genes may be constructed easily. In the conventional method of Tc mouse generation, microcell-mediated chromosome transfer (MMCT) has been used to transfer an intact chromosome, HAC, or MAC vector into mES cells (Fig. 1b). A HAC or MAC vector constructed in CHO cells is transferred to mES cells. Sometimes, a constructed HAC or MAC vector is transferred to mouse A9 cells prior to transfer to mES cells because a larger number of microcells form in mouse A9 cells than in CHO cells, enabling an increase in the transfer rate of MI-MAC to mES cells. However, the efficiency of MMCT is low because the cytotoxicity of polyethylene glycol results in cell damage and the loss of viable cells (10^−6^–10^−5^ per recipient cell) (Katoh et al. 2010).

To facilitate Tc mouse generation and increase the efficiency of the process, we have established mES cells containing the MI-MAC vector to directly insert a transgene by the site-specific recombination with integrases or FLPe recombinase in mES cells (without MMCT). We further investigated whether the site-specific recombination at five attachment sites was functional in mES cells and whether Tc mice could be generated following germinal transmission.

## Results

### Construction of MI-MAC mES cell lines

To establish mES cells containing the MI-MAC vector, the MI-MAC vector in CHO cells was transferred to mouse A9 cells, then into the mES cells by two rounds of MMCT (Fig. 1a, b). Twenty-five clones were obtained, and these mES cell clones were checked by genomic PCR, and FISH analysis for retention of the intact MI-MAC (Fig. 1c). Eight clones contained the intact MI-MAC (MI-MAC mES cell lines 1–8). Next, to evaluate the pluripotency of MI-MAC mES cell lines 1–8, these mES cells were aggregated with ICR eight-cell stage embryos to generate chimeric mice. Characteristics of MI-MAC mES cell lines are shown in Table 1. Chimeric mice derived from all MI-MAC mES cell lines were produced. To evaluate whether MI-MAC mES cell lines contribute to the germline, chimeric mice were mated. F1 mice were produced from female chimeric mice derived from MI-MAC mES cell lines 4 and 5, and were genotyped (Table 1). The germline transmission (GT) rate of female F1 MI-MAC Tc mice derived from MI-MAC mES cell line 4 and 5 was 45.3 and 57.1 %, respectively. We used MI-MAC mES cell line 4 for all subsequent experiments.

**Table 1:** Efficiency of MI-MAC chimeric mouse generation

ES lines	Sex of ES cells	Transferred embryos	Total offspring	Chimeric mice/transferred embryo (%)	Germline transmission^a^
1		99	25	8.1	No
2		42	2	2.4	N/A
3		48	2	2.1	N/A
4		139	34	22.3	Yes
5		48	8	12.5	Yes
6		48	9	8.3	N/A
7		48	15	29.2	N/A
8		381	59	13.4	N/A

^a^Germline transmission by natural mating

### Site-specific recombination with MI-MAC mES cells by PhiC31, R4, TP901-1 and Bxb1 integrase and FLPe recombinase

**Fig. 2:**
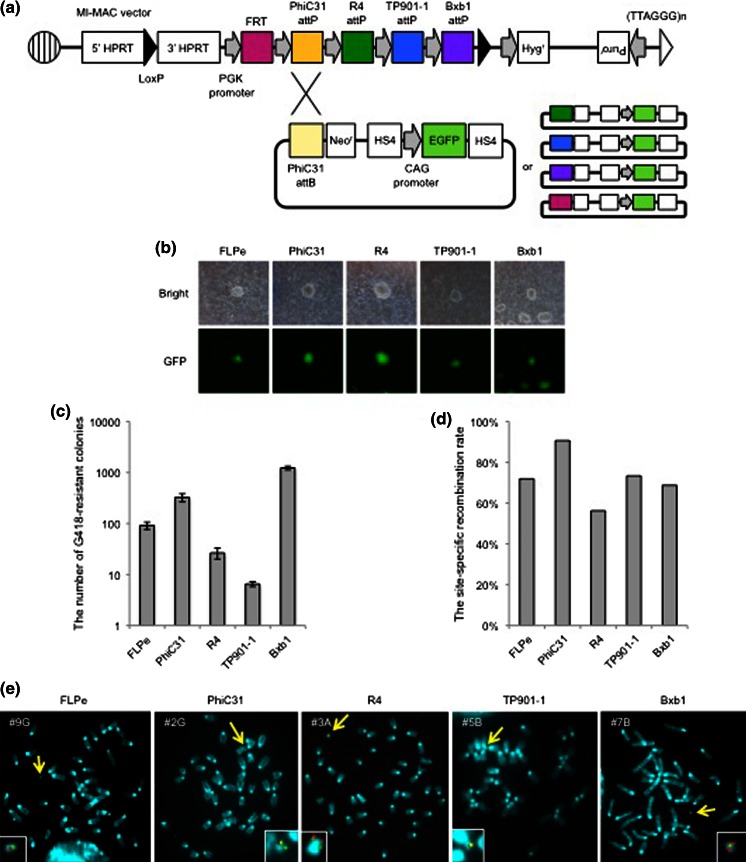
EGFP gene insertion into the MI-MAC in mES cells by FLPe recombinase, PhiC31, R4, TP901-1, or Bxb1 integrase. **a** Site-specific recombination of the EGFP gene by PhiC31, R4, TP901-1, or Bxb1 integrase or FLPe recombinase in MI-MAC mES cells. **b** EGFP expression in G418-resistant colonies electroporated with FRTneo-EGFP, PhiC31neo-, R4neo-, TP901-1neo-, or Bxb1neo-EGFP and the corresponding integrase or recombinase expression plasmid. The magnification is 4× and exposure time was 400 ms (GFP). **c** Numbers of G418-resistant colonies by FLPe recombinase, PhiC31, R4, TP901-1, or Bxb1 integrase. The data were corrected for average colony numbers (n = 2). **d** Site-specific recombination rate of FLPe recombinase, PhiC31, R4, TP901-1, or Bxb1 integrase by genomic PCR. The number of PCR positive clones of FLPe recombinase, PhiC31, R4, TP901-1 and Bxb1 integrase was 23, 29, 18, 11 and 22, respectively (n = 32; FLPe, PhiC31, R4, Bxb1, n = 15; TP901-1). **e** FISH analysis of 9G, 2G, 3A, 5B and 7B mES cell clones with the EGFP gene inserted by FLPe recombinase, PhiC31, R4, TP901-1 and Bxb1 integrase. Biotin-labelled GFP expression vector (*green*) and digoxigenin-labelled MI platform (*red*) were used to detect the GFP gene and the MI-MAC, respectively. The *inset* shows an enlarged MI-MAC vector with GFP inserted (*arrow*)

To determine whether site-specific recombination could be achieved with MI-MAC mES cells, a plasmid carrying the EGFP gene and an attachment site (FRTneo-EGFP, PhiC31neo-, R4neo-, TP901-1neo-, or Bxb1neo-EGFP) and the corresponding recombinase expression plasmid were co-transfected in MI-MAC mES cell line 4 by electroporation (Fig. 2a). Integrase- and FLPe-mediated site-specific recombination in MI-MAC mES cells was evaluated by G418-resistant colony formation, GFP expression in each colony, and PCR assay. The average numbers of G418-resistant colonies for each recombination using FLPe, PhiC31, R4, TP901-1 and Bxb1 were 92, 352, 27, 7 and 1229 colonies per electroporation, respectively, and almost all of these G418-resistant colonies expressed GFP (Fig. 2b, c). The frequency of site-specific recombination was confirmed by genomic PCR analysis at the 5′ junction region. PCR analyses using 5′ junction primers showed that the rates of site-specific recombination by FLPe, PhiC31, R4, TP901-1 and Bxb1 were 71.9, 90.6, 56.3, 73.3 and 68.8 %, respectively (Fig. 2d). Flow cytometric analysis showed that the population of EGFP-positive cells in the clones was over 95 % (Supplemental Figure 1). FISH analyses showed that the EGFP gene was inserted into the MI-MAC of FLPe-, PhiC31-, R4-, TP901-1- and Bxb1-expressing cells, and was not integrated into the host genome (Fig. 2e). These results demonstrated that a gene of interest could be inserted in MI-MAC mES cells using integrases or FLPe recombinase.

### Generation of Tc mice containing CAG-ELuc/MI-MAC

**Fig. 3:**
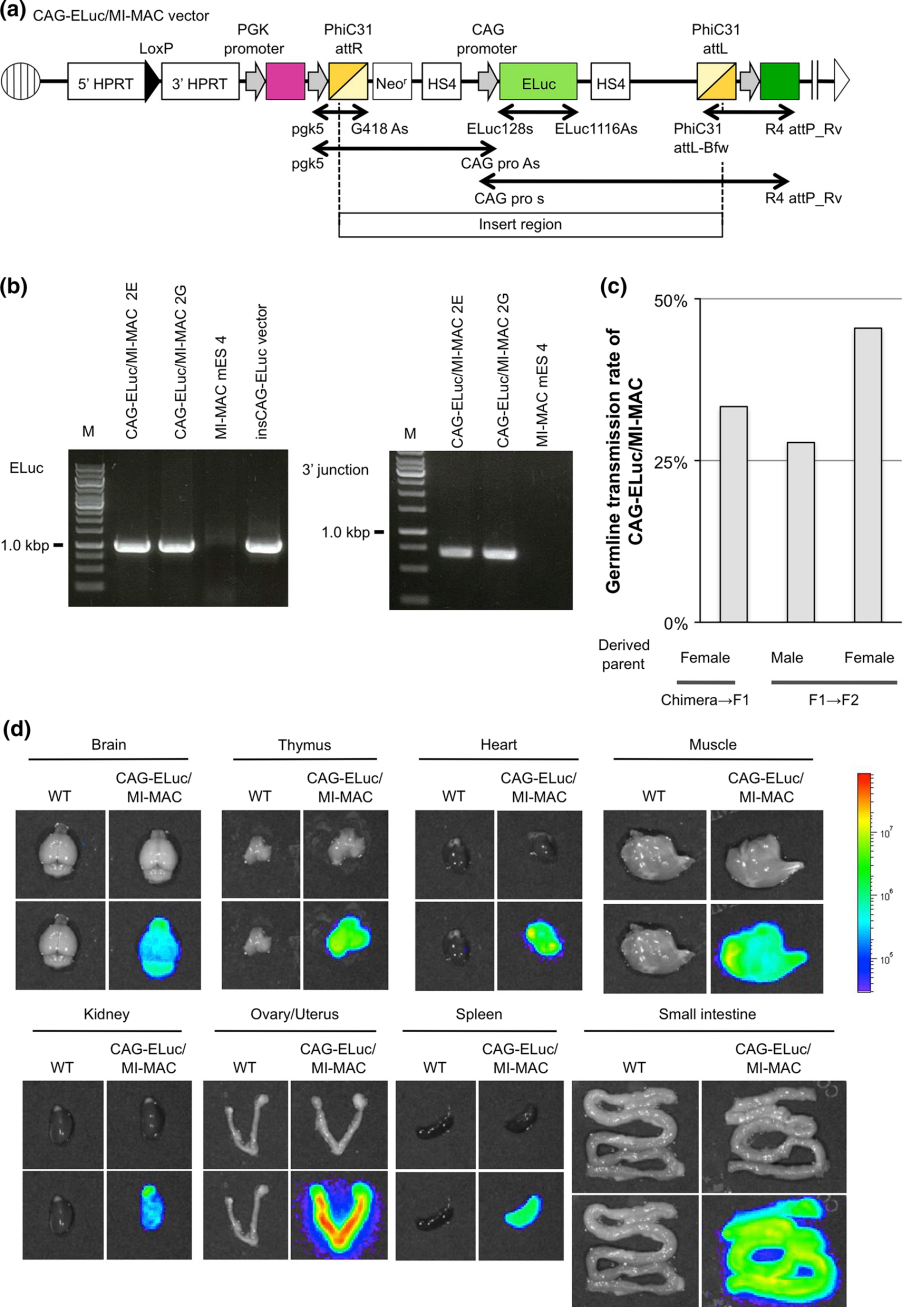
Characterisation of MI-MAC mES cells carrying the ELuc gene. **a** Map of MI-MAC ubiquitously expressing ELuc. *Two-headed arrows* show PCR primers used for confirmation of the ELuc gene insertion at phiC31 site on MI-MAC. **b** The genomic PCR at the ELuc gene and 3′ junctions of the MI-MAC in MI-MAC mES cell line 4. **c** Germline transmission rate of CAG-ELuc/MI-MAC Tc mice. The rate represents the ratio of CAG-ELuc/MI-MAC Tc mice to total offspring by mating with C57BL/6J. **d** Ex vivo bioluminescence imaging of the CAG-ELuc/MI-MAC Tc mouse tissues. The exposure time was 5 s

To confirm that MI-MAC mES cells containing gene of interest contributed to the germline and that MI-MAC Tc mouse strains could be generated, we established MI-MAC Tc mice expressing ELuc ubiquitously. The insCAG-ELuc vector carrying the ELuc gene driven by the CAG promoter and the PhiC31 integrase expression vector were co-electroporated into MI-MAC mES cell line 4. Nineteen randomly selected clones were used for the following analyses. ELuc was expressed in 15 of 19 clones (data not shown). In four randomly selected ELuc-positive clones, genomic PCR analyses using 5′ junction primers showed that the insCAG-ELuc vector was inserted at the PhiC31 site in MI-MAC (data not shown). Additional PCR analyses using ELuc gene primers, 3′ junction primers, and two long PCR primers showed that two mES cell clones (2E and 2G) of the four clones were positive (Fig. 3a). Further PCR analyses showed that the PhiC31 integrase cassette did not remain in the genome of the 2E and 2G mES cell clones (data not shown). Moreover, chimeric mice derived from 2E and 2G mES cell clones gave birth to offspring carrying MI-MAC with the ELuc gene. Therefore, the 2G and 2E mES cell clones had pluripotency with the ability to generate chimeric mice and the next generation.

The GT rate of CAG-ELuc/MI-MAC from female chimeric mice derived from 2G to F1 was 33.3 %, and from male and female F1 Tc mice to the next generation was 27.8 and 45.5 %, respectively (Fig. 3c). To demonstrate ELuc activity in vivo, Tc mice with CAG-ELuc/MI-MAC were examined by in vivo and ex vivo bioluminescence imaging. The whole body of the Tc mice emitted light ubiquitously (data not shown). The ELuc gene was expressed in all examined tissues of the Tc mice (Fig. 3d). The expression level of ELuc was different in each tissue, which was similar to a previous report (Kazuki et al. 2013b). Therefore, the gene expression level may be dependent on CAG promoter activity. Thus, it was possible to generate a Tc strain by direct electroporation of a target vector into MI-MAC mES cells.

## Discussion

In this study, we generated MI-MAC mES cell lines to create a simplified and efficient strategy for Tc mouse generation. We demonstrated that some MI-MAC mES cell lines had the potential to generate chimeric mice and could contribute to GT (Table 1). The site-specific recombination rates using one of four integrases or FLPe recombinase were 56.3–90.6 % in the MI-MAC mES cells (Fig. 2d). This site-specific recombination using MI-MAC mES cells was more effective than targeted homologous recombination (Soriano 1999). PhiC31 integrase was used for single-copy transgene insertion into predetermined loci by pronuclear microinjection (Tasic et al. 2011). If a gene of interest is inserted by microinjection into fertilised eggs derived from MI-MAC Tc mice without using MI-MAC mES cells, the method may be more efficient to generate Tc mice.

Site-specific recombination by PhiC31 integrase was more effective than that of others in MI-MAC mES cells, which was consistent with similar experiments using CHO cells containing MI-HAC (Yamaguchi et al. 2011). Five different circular vectors can theoretically be inserted into the MI-MAC of mES cells using a simple sequential transfection method, although five selection markers would be required. In a previous study, a large genomic P1 phage-derived artificial chromosome (PAC) vector was inserted to the HAC vector successfully (Kazuki et al. 2008). Thus, PAC vectors may also be inserted into the MI-MAC at the same or at a higher efficiency, which we will examine in a future study. To avoid the use of many selection markers, drug resistance genes can be disrupted by genome editing technology. Recently, Suzuki et al. reported that three target genes were simultaneously integrated on a HAC by Cre recombinase and PhiC31 and Bxb1 integrases in CHO cells (Suzuki et al. 2014). Although this system can be applied to mES cells, the disruption of *Hprt* gene would be required in the mES cells. Genome editing with site-specific nucleases such as Zinc-finger nucleases, transcription activator-like effector nucleases and clustered regulatory interspaced short palindromic repeat/Cas-based RNA-guided DNA endonucleases, has been previously used to modify the endogenous genome of several species (Geurts et al. 2009; Sung et al. 2013; Wang et al. 2013). A combination of Tc technology via MI-MAC mES cells and KO technology via genome editing will be useful for generation of humanised mice.

The GT rate of the MI-MAC was dependent on the sex of the founder. The GT rate of the MI-MAC derived from male founders was lower (20–30 %) than that derived from female founders (40–50 %), which was comparable to that of Tc mice containing the MAC1 (Kazuki et al. 2013b). To explain the different GT rates of mammalian artificial chromosomes between males and females, one hypothesis cites sex differences in meiotic division (Mee et al. 2003). To overcome the subfertility of male Tc mice, reproductive assistance by intracytoplasmic sperm injection and round spermatid injection may be useful (Mizutani et al. 2005).

We generated Tc mice that ubiquitously expressed ELuc using CAG-ELuc/MI-MAC mES cells. Bioluminescence imaging (BLI) allows us to non-invasively visualise or determine the quantity of gene expression. ELuc produces a stronger bioluminescent signal than does firefly luciferase in vitro (Nakajima et al. 2010). Therefore, CAG-ELuc/MI-MAC Tc mice represent a useful bioresource for studies requiring highly sensitive BLI in vivo, such as kinetics analysis of luciferase expression or cell- and tissue-transplantation experiments. On the other hand, a KI approach using specific integration such as that for the *Rosa26* locus will enable the generation of stable transgenic mouse lines containing a desired gene. A study comparing the efficiency of a KI versus a MI-MAC approach using the same construct is required to evaluate the transgene expression level and stability. We plan to undertake such a study in the future. A simple method using MI-MAC mES cells to enable the transfer of desired genes could therefore allow efficient development of Tc mice carrying the inserts for humanised models and monitoring of human genomic functions by BLI.

## Materials and methods

### Vectors

PhiC31, R4, TP901-1, Bxb1 integrase and FLPe expression vectors were synthesised de novo and were driven by the CAG promoter. These codon sequences were optimised as previously described by Yamaguchi et al. (2011). Plasmid vectors used in the recombination assay were PhiC31neo-EGFP, R4neo-EGFP, TP901-1neo-EGFP, Bxb1neo-EGFP and FRTneo-EGFP. These EGFP vectors were based on the inspB4ins2 vector containing two HS4 dimers derived from pJC5-4 (a gift from Dr. G. Felsenfeld). The CAG promoter, EGFP open reading frame (ORF), and SV40 poly-A were ligated into a multiple cloning site between the HS4 dimers on inspB4ins2 (Ins2CAG-EGFP). The CAG promoter was derived from pCX-EGFP (a gift from Dr. Okabe, Osaka University, Japan), and the EGFP ORF and SV40 poly-A were derived from EGFP-N1 (Clontech, USA). The PhiC31neo-EGFP, R4neo-EGFP, TP901-1neo-EGFP, Bxb1neo-EGFP, or FRTneo-EGFP vectors were constructed by ligation of the ins2CAG-EGFP vector with relevant inserts digested from the pNeo-PhiC31 attB, pNeo-R4 attB, pNeo-TP901-1 attB, pNeo-Bxb1 attB, or pNeo-FRT vectors, respectively. The insCAG-ELuc vector (a gift from Dr. Nakajima, AIST, Japan) was used as the plasmid carrying the Emerald Luc (ELuc; TOYOBO, Japan) from the Brazilian click beetle *Pynearinus termitilluminans* (Nakajima et al. 2010) driven by the CAG promoter.

### Cell culture

CHO cells were cultured in F12 medium (Wako, Japan) supplemented with 10 % foetal bovine serum (FBS) (PAA Laboratories GmbH, Australia) and 1 % penicillin/streptomycin (Gibco, USA). Mouse A9 cells were cultured in Dulbecco’s modified Eagle’s medium (DMEM; Gibco) supplemented with 10 % FBS and 1 % penicillin/streptomycin. TT2 and TT2F mES cells were hybrids of C57BL/6 and CBA strains (a gift from Dr. S. Aizawa, RIKEN, Japan). TT2 and TT2F mES cells and MI-MAC mES cell lines were cultured in KnockOut DMEM (Gibco) supplemented with 5 % FBS, 15 % KNOCKOUT SR (KSR; Gibco), 1× minimum essential medium non-essential amino acids (Gibco), 1× GlutaMAX™-1 (Gibco), 1× nucleosides (Millipore, Germany), 55 µM 2-mercaptoethanol (Gibco), 2.0 × 10^6^ units/mL ESGRO mLIF medium supplement (Millipore), and 1 % penicillin/streptomycin.

### Mice

ICR mice (SLC Japan) were used to generate Tc mice. Tc mice were backcrossed to C57BL/6 mice (SLC Japan). All animal experiments were approved by the Institutional Animal Care and Use Committee of Tottori University.

### MMCT

MMCT was performed as described previously (Tomizuka et al. 1997). Briefly, the MI-MAC was transferred from CHO cells into TT2 or TT2F mES cells via mouse A9 cells by MMCT. Donor cells with the MI-MAC were treated with 0.05–0.1 µg/mL colcemid (Gibco) to form microcells. Microcells were collected by centrifugation and fused with recipient cells by polyethylene glycol (Wako, Japan). Hybrid cells containing the MI-MAC were selected in media containing 1 × HAT Supplement (Gibco) or 200 µg/mL Hygromycin B (Wako, Japan).

### FISH analysis

FISH analysis was performed by standard protocols (Tomizuka et al. 1997). Briefly, fixed cells containing the MI-MAC at metaphase or interphase were spread on slides, and hybridised with biotin-labelled and digoxigenin-labelled probes in a nick translation mix (Roche, Germany). Chromosomal DNA was counterstained with 4′,6-diamidino-2-phenylindole (Southern Biotech, USA). Images were captured with ISIS (Carl Zeiss, Germany).

### Electroporation

MI-MAC mES cell line 4 was cultured to subconfluence. Before electroporation, MI-MAC mES cells were collected and plated on a gelatine-coated cell culture dish without feeder cells for 1 h, followed by recollection of cells in suspension. Plasmid vector carrying the gene of interest (8.5 µg) was co-transfected with the representative integrase/recombinase expression vector (3 µg) into 5.0 × 10^6^ MI-MAC mES cells per sample with an Amaxa™ mouse ES Cell Nucleofector™ Kit (Lonza, Switzerland). Forty-eight hours after transfection, these mES cells were selected in 75–150 µg/mL G418 for 1 week, and G418-resistant colonies were counted and GFP expression was observed using an NIS-Elements D 3.2 (Nikon, Tokyo, Japan).

### Flow cytometry

To determine the ratio of EGFP-positive cells, mES cells transfected with the EGFP gene by recombinase or integrase were collected, stained with DRAQ7 dye (Beckman Coulter, USA) to remove inactive cells, and then analyzed by a Galios flow cytometer (Beckman Coulter).

### Genomic PCR analysis

Genomic DNA from cells or tails of mice was extracted using a genomic extraction kit (Sigma, USA) or KAPA Express Extract DNA Extraction kit (Kapa Biosystems, USA). PCR analyses were performed using a standard protocol. To genotype MI-MAC Tc mice, hyg57s and hyg899As primers were used. To check site-specific recombination, pgk5 and CAG pro As primers were used. To check the insertion of the ELuc gene in the PhiC31 attachment site on the MI-MAC, ELuc128s and Eluc1116As primers, pgk5 and G418 As primers, pgk5 and CAG pro As primers, CAG pro S and R4 attP_Rv primers, and PhiC31 attL-Bfw and R4 attP_Rv primers were used (Fig. 3a). To genotype CAG-ELuc/MI-MAC Tc mice, ELuc128s and Eluc1116As primers were used. These primer sequences are listed in Supplementary Table 1.

### Tc mouse generation

MI-MAC mES cell lines or CAG-ELuc/MI-MAC mES cell lines were cultured to subconfluence. ICR female mice were superovulated with 5 IU pregnant mare’s serum gonadotropin and 5 IU human chorionic gonadotropin and mated with ICR male mice. Embryos at the two-cell stage were collected from mated female mice and cultured in KSOM medium at 37 °C under 5 % CO_2_. Eight-cell stage embryos from which the zona pellucidae were removed by Tyrode’s acid were aggregated with collected mES cells and cultured overnight. Cultured blastocysts were transferred into uteri of day 2.5 pseudopregnant ICR female mice. Chimerism of resulting chimeric mice was determined by coat colour. Chimeric mice were mated with C57BL/6 Jms Slc for backcrossing.

### Ex vivo BLI

CAG-ELuc/MI-MAC Tc mice were injected intraperitoneally with 150 mg/kg d-luciferin (TOYOBO, Japan). Ten minutes after luciferin injection, the major tissues were promptly excised from euthanized CAG-ELuc/MI-MAC Tc mice and imaged by IVIS Lumina imaging system (Xenogen).

## Electronic supplementary material

Supplementary material 1 (DOCX 1307 kb)
