# Supplementary material for: Mouse embryonic stem cells with a multi-integrase mouse artificial chromosome for transchromosomic mouse generation
Source: Transgenic Res. 2015 Jun 9;24(4):717–27. doi: 10.1007/s11248-015-9884-6 (PMC4504986; doi:10.1007/s11248-015-9884-6)
Supplement: Supplementary file 1 — Supplementary material 1 (DOCX 1307 kb) [file 11248_2015_9884_MOESM1_ESM.docx]

**Mouse embryonic stem cells with a multi-integrase mouse artificial chromosome for transchromosomic mouse generation**

Yuki Yoshimura^a, b^, Kazuomi Nakamura^a^, Takeshi Endo^c^, Naoyo Kajitani^d†^, Kanako Kazuki^d^, Yasuhiro Kazuki^b,d^, Hiroyuki Kugoh^b,d^, Mitsuo Oshimura^d,*^, and Tetsuya Ohbayashi^a,*^

^a^Division of Laboratory Animal Science, Research Center for Bioscience and Technology, Tottori University, 86 Nishi-cho, Yonago, Tottori 683-8503, Japan

^b^Department of Biomedical Science, Institute of Regenerative Medicine and Biofunction, Graduate School of Medical Sciences, Tottori University, 86 Nishi-cho, Yonago, Tottori 683-8503, Japan

^c^Organizations for Tottori Industrial Promotion, Tottori, Japan

^d^Chromosome Engineering Research Center (CERC), Tottori University, 86 Nishi-cho, Yonago, Tottori 683-8503, Japan

Present address:

**†**Department of Experimental Animals, Interdisciplinary Center for Science Research, Organization for Research, Shimane University, 89-1 Enya-cho, Izumo, Shimane 693-8501, Japan

*Corresponding author:

Chromosome Engineering Research Center (CERC), Tottori University, 86 Nishi-cho, Yonago, Tottori 683-8503, Japan. Phone: +81 859 38 6212. Fax; +81 859 38 6210. E-mail: [oshimura@med.tottori-u.ac.jp](mailto:oshimura@med.tottori-u.ac.jp). (M. Oshimura)

Division of Laboratory Animal Science, Research Center for Bioscience and Technology, Tottori University, 86 Nishi-cho, Yonago, Tottori 683-8503, Japan. Phone: +81 859 38 6475. Fax; +81 859 38 6479. E-mail: [ohbayashi@grape.med.tottori-u.ac.jp](mailto:ohbayashi@grape.med.tottori-u.ac.jp). (T. Ohbayashi)

**Supplementary data**


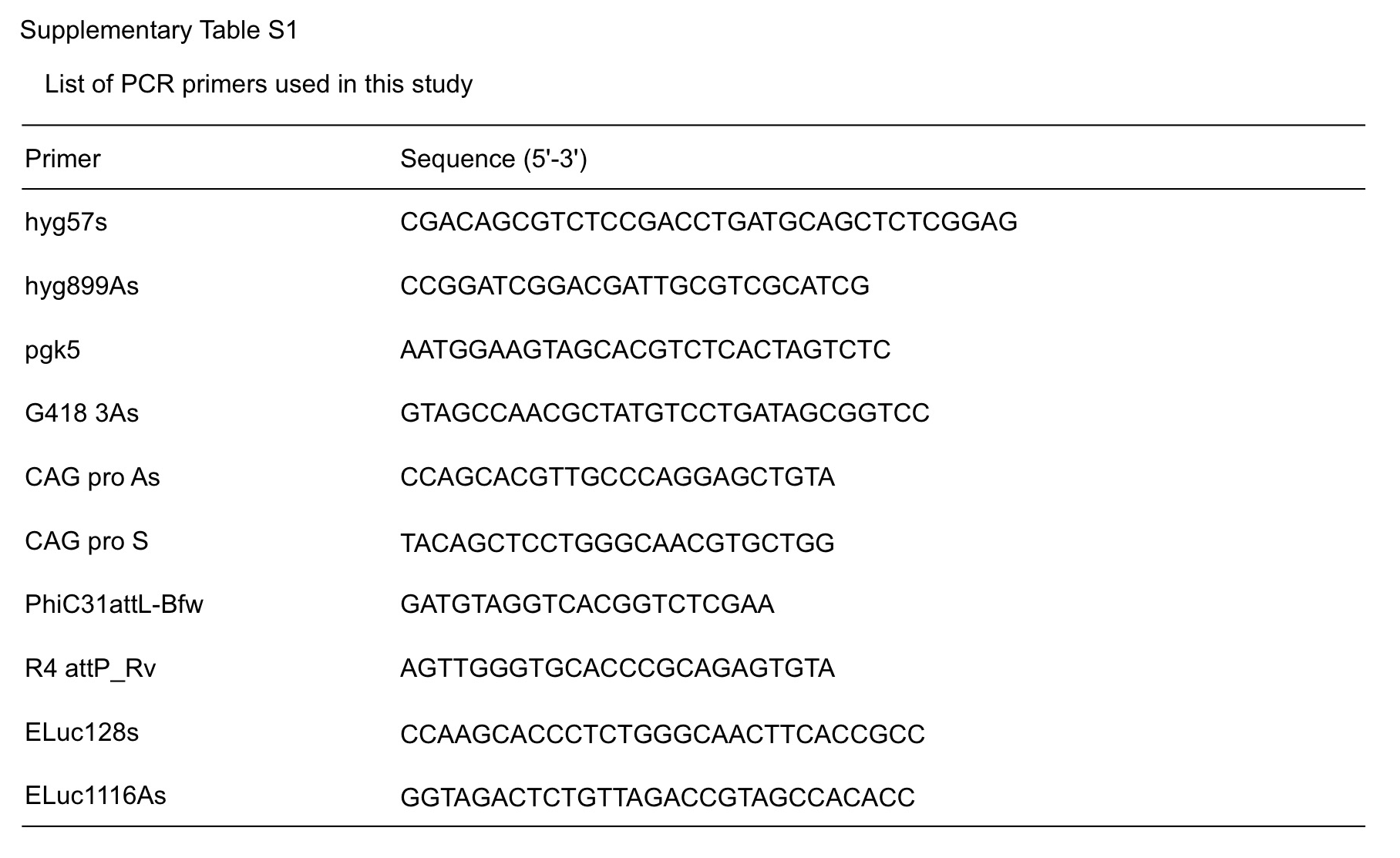


**Supplementary Table. 1 List of PCR primers used in this study**


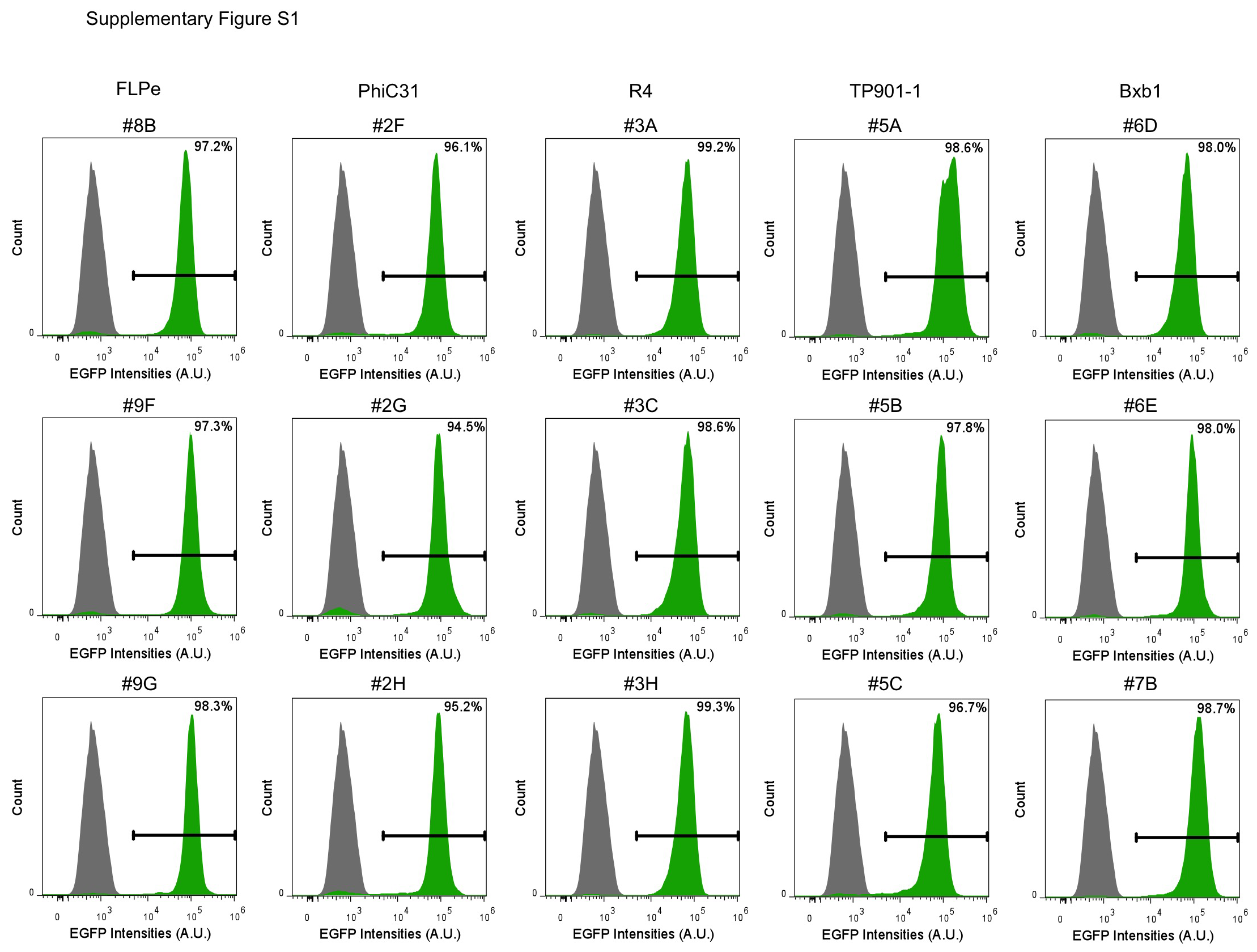


**Supplementary Fig. 1 The percentage of EGFP-positive cells**

Flow cytometry was used to analyze the percentage of EGFP-positive cells in mES cell clones with the MI-MAC inserted into the EGFP gene. Three clones each were analyzed for FLPe recombinase, and PhiC31, R4, TP901-1, and Bxb1 integrase. The clones used were as follows. FLPe: 8B, 9F, and 9G; PhiC31: 2F, 2G, and 2H; R4: 3A, 3C, and 3H; TP901-1: 5A, 5B, and 5C; Bxb1: 6D, 6E, and 7B. The control clone was MI-MAC mES 4.
